# Using Social Network Analysis to Strengthen Organizational Relationships to Better Serve Expectant and Parenting Young People

**DOI:** 10.1007/s10995-020-02992-6

**Published:** 2020-09-05

**Authors:** Amanda Purington, Erica Stupp, Dora Welker, Jane Powers, Mousumi Banikya-Leaseburg

**Affiliations:** 1 Bronfenbrenner Center for Translational Research, Cornell University, Ithaca, NY USA; 2 Bureau of Women, Infant & Adolescent Health, New York State Department of Health, Albany, NY USA; 3 Office of Population Affairs, U.S. Department of Health & Human Services, Rockville, MD USA; 4 Present Address: Heriot-Watt University in Edinburgh, Edinburgh, UK; 5 ACT for Youth, Bronfenbrenner Center for Translational Research, Cornell University, 35 Thornwood Drive Suite 200, Ithaca, NY 14853 USA

**Keywords:** Collaboration, Social network analysis, Young parents, Systems-level intervention

## Abstract

**Introduction:**

Expectant and parenting young people (young parents) need a range of supports but may have difficulty accessing existing resources. An optimally connected network of organizations can help young parents navigate access to available services. Community organizations participating in the Pathways to Success (Pathways) initiative sought to strengthen their network of support for young parents through social network analysis (SNA) undertaken within an action research framework.

**Method:**

Evaluators and community partners utilized a survey and analysis tool to map and describe the local network of service providers offering resources to young parents. Respondents were asked to characterize their relationship with all other organizations in the network. Following survey analysis, all participants were invited to discuss and interpret the results and plan the next actions to improve the network on behalf of young parents.

**Results:**

Scores described the diversity of organizations in the network, density of connections across the community, degree to which the network was centralized or decentralized, which organizations were central or outliers, frequency of contact, levels of collaboration, and levels of trust. Findings were interpreted with survey participants and used by Pathways staff for action planning to improve their network.

**Discussion:**

SNA clarified complex relationships and set service providers on a path toward optimizing their network. The usefulness of SNA to impact and improve a network approach to supporting young parents is discussed, including lessons learned from this project.

## Significance

Systems-level interventions can bring about broader, sustainable population impact. Such interventions typically require diverse individuals and organizations to partner with one another to achieve common goals. Functional relationships among strategically chosen partners are critical to such efforts but can be difficult to define, quantify, and monitor. This article presents a unique action research process to measure and understand network structure and relationships using social network analysis. Analysis results helped network members better understand existing organizational relationships, identify missing or weak relationships, and inform action planning to better serve their priority population.

## Introduction

Diverse supports are often required to meet the various needs of expectant and parenting young people (young parents) and their children; these include health and development, education, housing, child care, and tangible supports (Lachance et al. 2012; Pinzon and Jones 2012; Savio Beers and Hollo 2009). Though local community organizations may provide a wealth of resources to meet these needs, the organizations are often siloed, making identifying and accessing these resources daunting for youth (Lachance et al. 2012). Connecting to resources can be a long process involving multiple parties and often does not result in receiving services, either because the service is no longer available or the young parent has disengaged (Goldberg et al. 2018). A case management approach may help young parents navigate these resources but may not be cost effective or sustainable (Frieden 2010).

One way communities can address this challenge is to take a systems-level approach to improve service access and utilization that accounts for the multiple services designed to support young parents’ success (such as health and human services, education, child care, and tangible and social supports) and the interactions between those services (Leischow and Milstein 2006; Mabry et al. 2008). Collaboration is a systems-level strategy that enhances knowledge and resource sharing, capitalizes on expertise from multiple disciplines, and facilitates coordination and sustainability of services, allowing organizations to make a more substantial impact together than if they worked independently (Butterfoss and Kegler 2002; Butterfoss et al. 2008; Emshoff et al. 2007). By collaborating to coordinate their services, organizations can mitigate some of the barriers young parents face when accessing them. Systems-level approaches such as collaboration have been employed in other efforts to improve teen pregnancy prevention (Cassell et al. 2005; Chervin et al. 2005; Kegler et al. 2005; Mueller et al. 2017) and to support pregnant and parenting teens (Radcliff et al. 2018). Common outcomes of the collaborative efforts of such projects include resource sharing, increased community awareness and support, development of new resources or programs to meet identified gaps, reduced duplication of services, and policy changes to increase access to services (Chervin et al. 2005; Kegler et al. 2005; Mueller et al. 2017).

Pathways to Success (Pathways), a New York State Department of Health (NYSDOH) initiative funded by the federal Office of Population Affairs Pregnancy Assistance Fund, sought to improve health, educational, and family functioning outcomes for young parents using a collaborative approach. Implemented in three large, diverse urban communities in New York State, Pathways was composed of funded partnerships between a school district and a local community college (funded partners) that received support from Assets Coming Together for Youth Center for Community Action (ACT), a technical support center based at Cornell University. Referred to here as Communities A, B, and C, each is similar in terms of need because all have some of the highest numbers of young parents in the state. However, each community (informally defined in geographic terms as the city in which funded partners work) differed in how its network of resource and service providers worked together. Over the course of the initiative, ACT and NYSDOH collaborated with funded partners and other organizations in their communities in an action research process (Bradbury 2015) to foster connections between organizations, sustain networks over time, streamline access to resources, and promote awareness of services.

During Pathways planning, qualitative data gathered from young parents and service providers identified the need for better communication and coordination between community resources for young parents, reinforcing the need for collaboration (Purington et al. 2020). Collaborative approaches can be challenging to implement and evaluate because the explicit activities and outcomes are difficult to define and quantify (Frey et al. 2006; Woodland and Hutton 2012). Identifying and mapping these partnerships is one approach for evaluating collaborative activities (Woodland and Hutton 2012). Therefore, before relationships could be strengthened in the funded communities, organizations first had to understand the existing networks. Using social network analysis (SNA), programs gained a deep understanding of how all organizations interact and relate to each other as a network (whole-network functioning), beyond simple descriptions of a single organization’s connections to other service providers in the community. When conducted at the start of an initiative, an analysis of an organizational network can help identify not only existing relationships and key players but also key markers of whole-network functioning, such as the number and diversity of organizations connected to each other (breadth), the intensity of those relationships (depth), and the overall connectivity of the network (Varda et al. 2008). Depicting these relationships in social network maps can elucidate existing relationships between local organizations, identify missing relationships or weakly connected organizations, and inform strategic efforts to strengthen the organizational network. This article discusses lessons learned about conducting and utilizing SNA in an action research process, using Pathways as an example. We focus on sharing this approach and its potential to impact and improve a network of support for young parents.

## Method

This project took an action research approach, which is a collaborative process of data gathering, critical reflection, and planning to improve practice (Bradbury 2015; Koshy et al. 2011). To yield the most useful understanding and develop action plans, *participants* should interpret the context-specific data gleaned from such a process (Koshy et al. 2011; Palus and McGuire 2015). Pathways-funded partners were critical in this process because they were both part of the network *and* charged with strengthening relationships among network organizations. Because of these dual roles, the funded partners were involved in every step.

With support from ACT and NYSDOH, each community individually conducted the SNA, data interpretation, and action planning. First, the funded partners in each community jointly created a list of organizations serving young parents—including the ones with which at least one funded partner had a relationship or believed should be connected to this network, given its resources or its link to the priority population. The process thus combined actual and potential partners from the community’s system of support for young parents. Funded partners were encouraged to think broadly and consider nontraditional potential network members. Identified organizations represented a wide range of providers, including health care, social services, nonprofit community-based organizations, educational institutions, faith-based organizations, and statewide or national institutes that were active locally. The number of actual and potential network members identified in each community ranged from 16 to 40. All agencies served young parents or families, though that might not have been their primary population.

The Program to Analyze, Record, and Track Networks to Enhance Relationships (PARTNER tool; Visible Network Labs 2010) was used to develop the online survey and to collect and analyze survey data.^1^ With a focus on assisting collaboratives to understand and build relationships between organizations, the PARTNER tool is an easy-to-implement process for social network analysis. PARTNER consists of an online survey with validated questions for assessing the network (with the ability to customize questions) and an Excel-based macro to compute network and individual organization scores and create network maps. The final survey asked organization representatives to characterize their relationship with all other organizations in the network, including the presence/absence of a relationship (“select organizations/programs/departments with which you have an established relationship, either formal or informal”); frequency of contact; specifically defined level of collaboration (Frey et al. 2006); and value of and trust within the relationship. If one organization indicated a relationship with another organization, that was considered a connection; relationships could be uni- or bi-directional. Respondents were also asked to indicate their potential contributions to the network, potential outcomes of collaboration, how well community organizations in the network work together, and facilitators of collaboration. Funded partners reviewed the survey questions and suggested revisions and additions; this involvement increased their understanding of and buy-in for the SNA process. Because the survey asked about organizations’ relationships with each other and not information about individuals, Cornell University’s Institutional Review Board for Human Participants determined this project was not human participant research and did not require Institutional Review Board review.

Next, funded partners contacted representatives of network organizations, both actual and potential partners identified by the funded partners, to inform them of the purpose of the survey and encourage participation, emphasizing its relevance to the community. Nearly all of the individuals invited to take the survey were frontline staff or mid-level supervisors and had some connection to the funded partner staff. These representatives were encouraged to pass the survey to another organization representative if they believed another person would be better positioned to respond to the questions. A modest incentive ($15 Amazon gift card) was offered to individual participants for completing the survey and to acknowledge the time commitment. Respondents had approximately 1 month to finish the online survey. Based on their relevance to young parents, some organizations were included as potential network members on the survey, even though funded partners could not identify a specific representative to complete the survey. In some of these cases, email invitations to complete the survey were sent to general organizational contact email addresses; in other cases, no organizational representative was contacted to complete the survey. This decision likely decreased response rates but allowed participants to consider the larger community system of support when rating the network.

The PARTNER tool was used to compute structural signatures such as the breadth, density, degree centralization, and trust of the overall network in each community (see Retrum et al. 2013 for an overview). *Breadth* refers to the diversity of organizations in the network. One indicator of breadth is the range of potential resource contributions from each organization to the network (e.g., specific expertise, funding, volunteers, or connections to community leaders). *Density* refers to the degree to which members of the network are connected and is an indicator of overall cohesiveness. Density is computed as the proportion of ties (relationships between organizations) that exist in a network out of the total number of possible ties (Prell 2012; Retrum et al. 2013). *Degree centralization* is a whole-network index of the extent to which a few organizations in the network hold central positions; high scores indicate a few organizations are central, whereas low scores indicate more equally distributed connections (Retrum et al. 2013). The *trust* index is composed of ratings of perceived reliability (“How reliable is this organization?”), openness to discussion (“How open to discussion is this organization?”), and mutual support of mission (“To what extent does this organization’s mission include better serving expectant and parenting teen and young adults in [community]?”). These ratings were combined for a trust score for each organization and averaged for the overall network. Frequency of contact (yearly, quarterly, monthly, weekly, daily); level of collaboration (a continuum of less to more collaboration: networking, cooperation, coordination, coalition, collaboration, from Frey et al. 2006); and perceived benefits and costs of collaboration provide further insights into organizational relationships and network functioning. Qualities of relationships between individual organizations (such as frequency of contact and levels of trust and collaboration) can be depicted visually in network maps. The maps depicting the overall network help visualize whole-network scores such as density and degree centralization.

Although addressing similar issues, each community—and its organizations—has its unique history and context. Involving survey participants in data interpretation and action planning was essential to develop relevant and effectual strategies to strengthen the network. Funded partners in each community received a detailed SNA report, including whole-network scores (e.g., density, degree centralization, trust); perceptions of the benefits and challenges of collaboration; potential resource contributions; and scores of individual organizations’ relationships to other organizations in the network. Also included were maps of the greatest potential resource contribution from each organization, frequency of contact, and levels of collaboration. This information, except for individual organization network scores, was also shared when interpreting data, in what we refer to as “data dialogue” sessions, described below.

Once analyses and reports were completed for each community, the SNA results were discussed with network members. Representatives of the organizations that were invited to complete the SNA survey were asked to participate in their community’s data dialogue session, where findings were discussed and interpreted by a team of evaluators, funded partners, and other key community stakeholders (Koshy et al. 2011; Palus and McGuire 2015).

Large versions of network maps, displaying the number of relationships, frequency of contact, and level of collaboration between all members, were presented in data dialogue sessions. Following a short presentation on the Pathways project and its focus on strengthening community systems and an overview of SNA and how to interpret the maps, participants were invited to review and discuss the results. Participants were encouraged to identify their organization in each map, look at the larger network, and consider the relationships depicted by discussing the following questions:

1. What stands out? What do you notice about the whole map?
2. What do you notice about your organization and its relationship to other organizations?
3. Which organizations are “key players”?
4. Which relationships need to be developed?

Discussion facilitated by ACT staff guided organization representatives as they interpreted the maps, considered these questions, discussed context, and brainstormed action plans to improve the relationships between organizations. Each session ended with a review of the actions planned and a discussion of next steps for the network. Because our intent is to illustrate the usefulness of SNA within an action research project to strengthen community networks and not to share the full SNA results, we present select findings from the SNA as examples that sparked key discussions and subsequent action planning. We hope this example of using SNA results to inform program planning and our lessons learned will be useful to other initiatives working to strengthen community systems of care.

## Selected Results

Table 1 presents survey response rates, number of potential network members, and structural signatures for each community network. Density scores are low across all three communities, indicating low cohesiveness in these potential networks and a great opportunity to build relationships between network members. The large range in degree centralization scores suggests these networks vary in the extent to which a few key organizations connect all other members. For example, Community C has a few central organizations with connections to most other organizations in the network. In contrast, Community A has more equally distributed connections. All three networks were rated high in trust, indicating that despite wide differences in density and degree centralization, existing relationships in these community systems of care are rated as highly trusting. These differing score patterns across communities suggest each network would require different improvement strategies.

**Table 1:** Network size, response rate, and whole-network scores for each community

Community	Response rate (# organizations in the actual and potential network)^a^	Density	Degree centralization	Trust
Community A	75% (16)	18.6%	34.4%	77.8%
Community B	51% (39)	23.2%	44.9%	68.7%
Community C	50% (40)	30.4%	70.6%	76.9%

^a^Representatives from organizations in the network within each community were asked to complete the network survey. Funded partners did not have contacts for all potential network members, decreasing the overall response rate

Potential resource contributions of network members across all three communities, presented in Table 2, indicate a range of possible contributions, with many organizations able to provide expertise and connections to others in the network and beyond, a willingness to engage in discussion and provide feedback to network members, and an ability to engage the priority population. Although few organizations could offer funding or in-kind resources, at least one in each community was able to do so.

**Table 2:** Frequency of potential resource contributions from network members in each community as an indicator of network breadth

Potential resource contribution	Community A	Community B	Community C
Specific health expertise or services	9	9	13
Expertise or services other than in health	6	6	8
Access to reach and engage the target population	8	14	13
Funding	2	3	1
In-kind resources	5	7	6
Paid staff	4	3	3
Volunteers or volunteer staff	3	4	3
Data resources including data sets, collection, or analysis	4	6	4
Discussion with or feedback for other network members	9	14	13
Connections to other organizations or leaders in our community	10	12	15
Facilitation/leadership	6	10	9
Advocacy	5	10	10
IT/web resources	3	2	1

The network consisted of 16 members in Community A, 39 members in Community B, and 40 members in Community C. Respondents could select more than one resource contribution, so the total number of resources exceeds the total number of members in each network

**Fig. 1:**
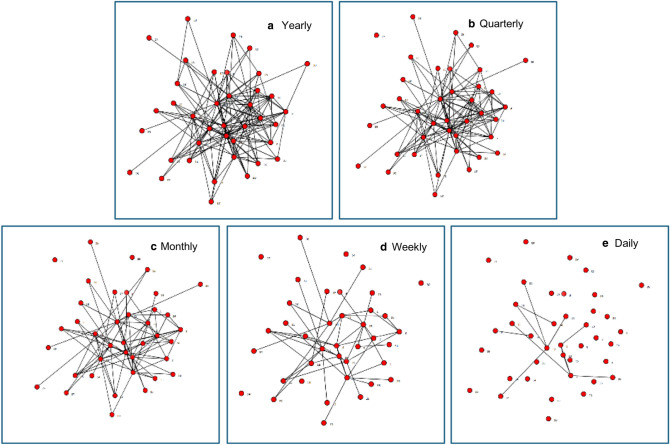
Frequency of contact for Community B (at least)

Frequency of contact between organizations in a network provides further insight into network functioning. The network members in Community B have relatively infrequent communication (as depicted in Fig. 1).

In this example, the “Yearly” frequency of communication reveals all network members have at least annual contact with other network members. However, several of the organizations on the periphery of the network are connected to only one other network member, which means the overall network is not very dense—many of the potential relationships are unrealized. As the maps of increasing contact frequency depict, the overall network appears much less connected as measures of contact increase in frequency. This pattern reinforces characteristics of Community B presented in Table 1; network members are loosely connected in that most are connected to at least one other organization with at least yearly contact, but there is room to build relationships with additional organizations in the network and increase frequency of contact if appropriate for the network’s functioning.

Table 3 presents the driving questions for data dialogue discussions, examples of observations that were discussed in response to each question, and examples of initial action planning steps relevant to the observations. Data dialogue participants in Community B noticed the lack of density in their network: most contact was between members at quarterly or yearly intervals (see Fig. 1). This low level of connection was also apparent in levels of collaboration, with most collaborative effort only at the networking or cooperation level (data not shown; Frey et al. 2006). Through discussion, network members brainstormed possible ways to enhance communication and collaboration without increasing the burden on members’ limited time by creating yet another meeting to attend. They ultimately decided to set aside time in an existing quarterly meeting that brought most network members to the same table to discuss the needs of young parents and learn more about the resources and services each organization provides. This would take the form of a “round robin” style of facilitated networking during 15 minutes of this regular meeting, encouraging network members to interact with individuals they did not already know, helping to ensure new connections were established, and promoting discussion about the needs of young parents.

**Table 3:** Discussion questions from facilitated data dialogue sessions, example observations that arose during discussions, and subsequent action planning

Data dialogue discussion question	Example community	Example data dialogue observation	Action plan
What stands out? What do you notice about the whole map?	B	This is not a dense network Much of the contact between network members is very infrequent (see Fig. 1)	Relationships need to be enhanced but members do not want more frequent meetings Communication can improve by incorporating discussion of young parents’ needs into existing meetings
What do you notice about your organization and its relationship to other organizations?	B	Most collaboration occurs at less intensive levels (networking, cooperation)	More intensive levels of collaboration are not always needed for effective relationships The Pathways project is an opportunity for network members to identify the most effective intensity of collaboration needed
Which organizations are ‘key players’?	C	This is a dense network; most organizations know each other (see all maps in Fig. 2) High degree centralization means a small number of organizations are central to the network Overall contact is infrequent and collaboration occurs at a lower intensity meaning fewer “key players.” There are some organizations on the periphery, connected to few or no other organizations	A connection should be established and then strengthened with organizations on the periphery of this network Future conversations will focus on developing collaboration
Which relationships need to be developed?	C	Peripherally connected organizations are easy to identify in this map, which would not be so apparent with a table of numbers indicating relationship strength The organization key enabled participants to easily identify which node on the maps represented which organization	Participants discussed ways to connect with one peripheral organization (a phone-based community referral provider), including leveraging existing relationships between this peripheral organization and other network members to strengthen this organization’s connection to the overall network

**Fig. 2:**
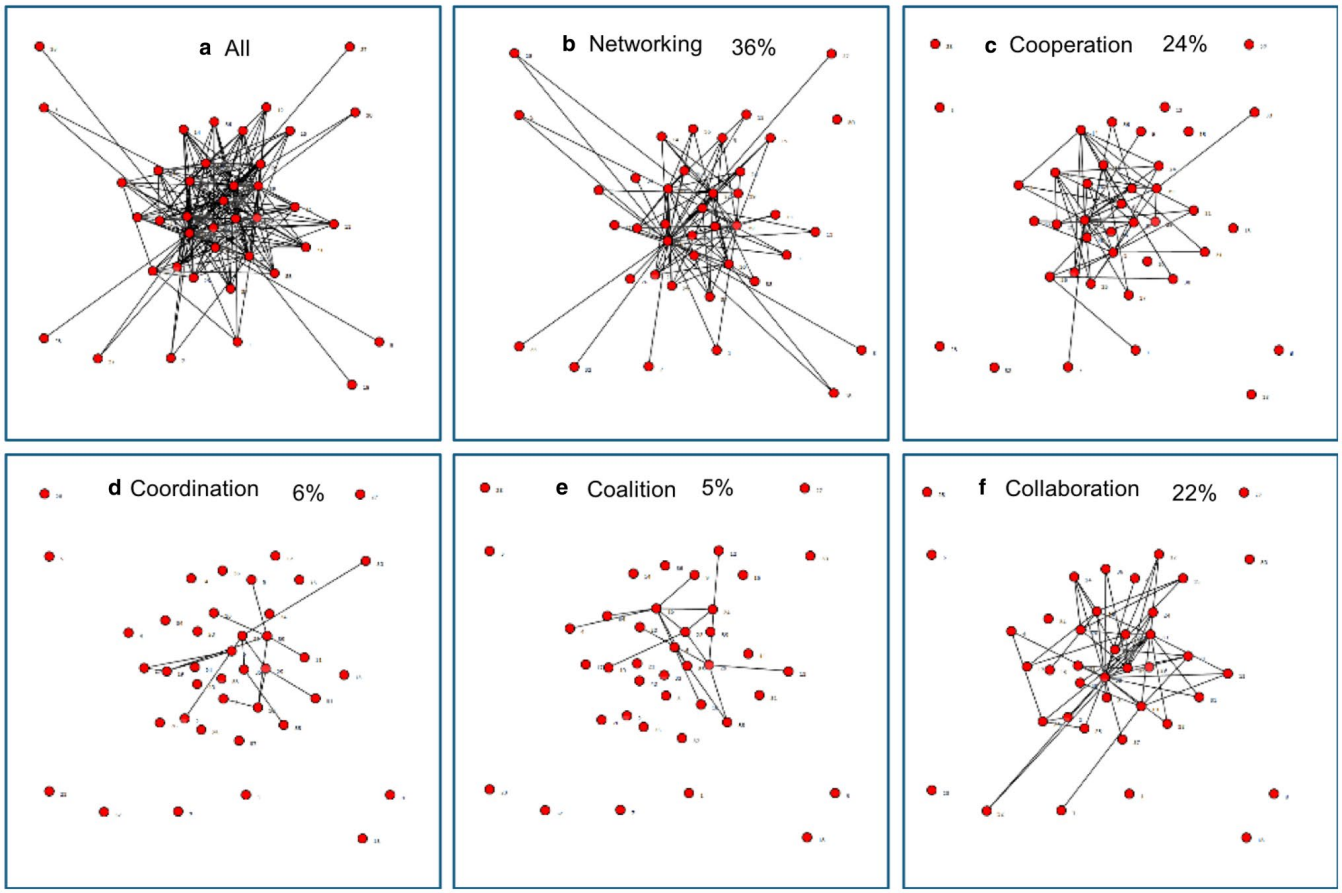
Level of collaboration for Community C. Levels of collaboration, a scale from less to more collaboration, are defined as follows (Frey et al. 2006): *Networking* involves awareness of the other organizations, loosely defined roles for working with the group of organizations, and little or infrequent communication with other organizations; all decisions are made independently of other organizations and their goals. *Cooperation* includes things like providing information to other organizations, having somewhat defined roles for working with the group of organizations, and having formalized communication with other organizations; however, all decisions are made independently of other organizations and their goals. *Coordination* includes things like sharing information and resources with other organizations; having defined roles for working with the group of organizations; having frequent, formalized communication with other organizations; and sharing some decision making with other organizations. *Coalition* involves sharing ideas along with frequent and prioritized communication with other organizations; all members have a vote in decision making regarding the common goals and activities of the group. *Collaboration* means that members belong to one system, frequent communication is characterized by mutual trust, and consensus is reached on all decisions regarding the common goals and activities of the group. Percentages in panels **b**–**e** indicate the proportion of all relationships in the network reported to be at that level of collaboration

Responses to maps in Community C illustrate the value of visually depicting organizational relationships. At the start of this community’s data dialogue session, one participant said, “Yup, that’s us!” The “All” collaboration map in Fig. 2 shows a tightly connected ball surrounded by a few more distally connected organizations, a depiction of their network that resonated with data dialogue participants.

However, these tight connections did not translate into truly working together. Most of the contact was infrequent and most of the collaboration occurred at a lower intensity. Figure 2 illustrates this shift; as levels of collaboration increase in intensity from networking to coalition, the proportion of organizational relationships functioning at that level decrease. However, this trend is reversed for the highest level of collaboration, further reinforcing the idea of the network in Community C is highly centralized. For those few organizations at the center of the network, communication is frequent and their level of collaboration is high. In its data dialogue discussion, Community C focused on ways to bring the peripherally connected organizations closer to the network. For example, by identifying specific organizations on the maps, data dialogue participants realized a key potential partner—a phone-based community referral provider—was only connected to two other organizations in the network and these relationships were only at the networking level. Enhancing relationships with this organization could lead to greater access to resources and supports for young parents. By looking at the network maps, data dialogue participants were able to determine which organizations had relationships with this outlier and to discuss a strategy to leverage their mutual contacts to establish a direct relationship with this referral provider.

## Discussion

The Pathways initiative aimed to improve health, educational, and family functioning outcomes for young parents by fostering connections between community organizations, an approach used in public health (Cassell et al. 2005; Chervin et al. 2005; Kegler et al. 2005; Mueller et al. 2017; Radcliff et al. 2018). SNA is a powerful tool to visualize complex relationships among network members and reveal characteristics of networks from a new perspective, an effort that can potentially improve relationships between community organizations. Although optimizing the level of collaboration and frequency of contact was a goal of Pathways, simply increasing the frequency of contact or level of collaboration between all organizations was not. Data dialogue participants discussed appropriate levels of contact and collaboration based on network needs.

Coupling SNA with an action research approach, in which respondents interpret and use results to improve their network functioning on behalf of young parents, is a strong combination. Through this process, Pathways-funded partners and their network members identified strengths and weaknesses of their networks, discussed ideal network functioning, and brainstormed action steps. The purpose of this paper is to share this process so others can learn from our experiences planning and conducting SNA, using results for program planning, and involving key stakeholders rather than to disseminate our specific project findings. The selected results presented here illustrate some of the network characteristics that sparked the most discussion in each community and the ways network members used information to develop plans to strengthen their community system of support.

Pathways-funded partners in Communities A, B, and C actively took part in the action research process, identifying members of their networks, encouraging participation in the survey that enabled the SNA, and convening network members in data dialogue sessions. Their participation was essential to make this project more locally relevant and useful both to Pathways-funded partners and other organizations in their networks. Funded partners’ involvement ensured the local relevance of the survey questions, likely increased survey completion, and facilitated network member discussion of the SNA results. Subsequent Pathways efforts were informed by the SNA results and data dialogue discussions.

The visual nature of the SNA maps clarified complex concepts (such as network density and degree centralization) in ways that allowed network members to see both the “big picture” of the network and the relationships between individual organizations. By facilitating discussion about these maps and network characteristics, network members were able to review and discuss SNA results, yielding rich and context-specific interpretations that were unlikely to surface if a lengthy report of SNA findings had merely been disseminated to network members. By engaging network members in a discussion of SNA results, including maps and network relationship qualities, they identified their networks’ strengths and weaknesses and discussed ways to build or improve them. Even the process of jointly interpreting network data led to building connections between organizations, a key objective of Pathways. The ideas generated to optimize relationships and facilitate connections came from network members, increasing the likelihood they would be actionable and impactful steps to improve the network of support for young parents in the unique contexts of their communities.

Each community developed specific, actionable steps to strengthen its network during the data dialogue sessions. These ideas ranged from committing to strengthen relationships between loosely connected organizations to connecting with organizations on the periphery of the network. By strategically increasing the number and optimizing connections between diverse network members, these efforts enhanced the chance that a young parent accessing resources offered by one network member would be connected with other organizations in the network, increasing young parents’ access to a wide array of service providers.

Other projects seeking to build and enhance organizational collaboration in communities as a way of increasing access to services may wish to use an approach similar to our action research SNA process. SNA is customizable to different subject areas and organizational contexts; the PARTNER tool makes this approach accessible without advanced experience with this methodology. An action research framework in which community partners participate in survey development, administration, and data interpretation increases investment in the process and the use of the results. The resultant action plans are likely to be meaningful, context relevant, and feasible.

Though this approach has significant advantages, it also brings challenges. For example, the developmental and participatory nature of the process made time management a challenge. Coordinating multiple players (funded partners, community organizations, and evaluators) across multiple stages of the process took longer than anticipated. Additionally, creating and sustaining buy-in at multiple levels is essential. Others using a similar process should allow for substantial time at the outset to build buy-in. Partner and network member involvement was critical to generating useful results and action planning; future applications of this approach should start this process early and plan for a longer and more flexible timetable.

Our team also weighed several influential decisions beginning the SNA. For example, our driving motivation was to gather data for program planning and to help stakeholders think about the big picture of their community system of support at the start of this initiative. As such, funded partners were encouraged to think broadly about *potential* network members in addition to organizations with which they already had a relationship. This ultimately decreased the survey response rate (because not all potential members had an identified point of contact), which lowered network density scores. It is unclear how depictions of the network would have changed had representatives from all organizations responded to the survey; perhaps different organizations would have emerged as key players and additional or unique resources could have been identified. However, the characteristics of the network revealed through these limited survey responses reflect the network as experienced by the very people funded to develop the network; identifying and addressing these limited connections was important. Visualizing connections resulted in rich discussions during data dialogues about organizations that were not but should be part of the network and potential ways to engage them.

Some evidence suggests improving community systems of care will result in better outcomes for young parents and their families (Bloxham 1997; Rosell et al. 2010). However, current relationships are the result of unique contextual dynamics in each community, and thus network members must be centrally involved in determining the strategies used to strengthen the network. Increasing network density, frequency of contact, or network diversity may not be the most important approaches to yield better outcomes for young parents in a particular community: there is no one-size-fits-all solution. Additionally, strong connections between community service providers may be necessary but insufficient to yield improved outcomes. Providers within these community systems of care may simply be overextended and unable to increase the number and quality of client interactions. Future research should explore the extent to which improving collaboration between service providers increases access to and use of services by young parents and explore the influencing role of contextual factors (such as inter-organizational relationships) in those improvements.

When community organizations work together at the systems level to achieve common goals, they may be able to make communitywide, sustainable impact. Such interventions typically require diverse individuals and organizations to partner with one another to achieve common goals. Functional relationships among strategically chosen partners are critical to such efforts but can be difficult to define, quantify, and monitor. Visualizing relationships between organizations through SNA and engaging network members in interpreting results in a “system self-examination” can help identify meaningful and feasible ways to strengthen the network and more effectively support the network members’ collective efforts.

## Footnotes

1 See https://www.visiblenetworklabs.com/partnertool for an overview of the PARTNER tool, which has been used in more than 2000 communities across all U.S. states and internationally in 40 countries.
